# Differences in Small Molecule Neurotransmitter Profiles From the Crown-of-Thorns Seastar Radial Nerve Revealed Between Sexes and Following Food-Deprivation

**DOI:** 10.3389/fendo.2018.00551

**Published:** 2018-10-15

**Authors:** Meaghan K. Smith, Utpal Bose, Masatoshi Mita, Michael R. Hall, Abigail Elizur, Cherie A. Motti, Scott F. Cummins

**Affiliations:** ^1^Genecology Research Centre, University of the Sunshine Coast, Maroochydore, DC, Australia; ^2^Center for Advanced Biomedical Sciences, TWIns Research Institute for Science and Engineering, Waseda University, Tokyo, Japan; ^3^Australian Institute of Marine Science (AIMS), Cape Ferguson, Townsville, QLD, Australia

**Keywords:** crown-of-thorns seastar, COTS, great barrier reef, neurotransmitter, metabolites, biosynthesis pathway

## Abstract

Neurotransmitters serve as chemical mediators of cell communication, and are known to have important roles in regulating numerous physiological and metabolic events in eumetazoans. The Crown-of-Thorns Seastar (COTS) is an asteroid echinoderm that has been the focus of numerous ecological studies due to its negative impact on coral reefs when in large numbers. Research devoted to its neural signaling, from basic anatomy to the key small neurotransmitters, would expand our current understanding of neural-driven biological processes, such as growth and reproduction, and offers a new approach to exploring the propensity for COTS population explosions and subsequent collapse. In this study we investigated the metabolomic profiles of small molecule neurotransmitters in the COTS radial nerve cord. Multivariate analysis shows differential abundance of small molecule neurotransmitters in male and female COTS, and in food-deprived individuals with significant differences between sexes in gamma-aminobutyric acid (GABA), histamine and serotonin, and significant differences in histamine and serotonin between satiation states. Annotation established that the majority of biosynthesis enzyme genes are present in the COTS genome. The spatial distribution of GABA, histamine and serotonin in the radial nerve cord was subsequently confirmed by immunolocalization; serotonin is most prominent within the ectoneural regions, including unique neural bulbs, while GABA and histamine localize primarily within neuropil fibers. Glutamic acid, which was also found in high relative abundance and is a precursor of GABA, is known as a spawning inhibitor in seastars, and as such was tested for inhibition of ovulation *ex-vivo* which resulted in complete inhibition of oocyte maturation and ovulation induced by 1-Methyladenine. These findings not only advance our knowledge of echinoderm neural signaling processes but also identify potential targets for developing novel approaches for COTS biocontrol.

## Introduction

The corallivore asteroid Crown-of-Thorns Seastar (COTS, *Acanthaster planci* species complex) is a keystone species within the natural biodiversity of coral reef ecosystems throughout the Indo-Pacific (1, 2). When present in low density, COTS consumption of hard coral does not outstrip overall coral growth rates (3). However, dramatic increases in population density, called outbreaks, typically lead to a significant loss of coral cover, ranging from 37% to over 99% (4–6), and of overall coral reef biodiversity, which in combination with other ongoing chronic stressors (e.g., climate change, cyclones) (4) are detrimental to coral reef ecosystems. The approach to controlling COTS outbreaks currently requires dive teams manually injecting a bile salt solution or poison with a drench gun directly into individual COTS of target populations on the reef, yet this has had a minor impact on overall numbers due to the large-scale populations and expanse of the Great Barrier Reef (GBR) (7). Further investigation at the molecular, biological, and chemical levels is required to determine exactly how COTS function, which could help to guide the development of novel methods for their biocontrol. The recent sequencing and annotation of the COTS genome (8) has made this more achievable. One area that requires further in-depth investigation for COTS, and echinoderms in general (e.g., sea cucumbers, sea urchins), is the mechanism(s) by which neural signaling by small molecule neurotransmitters (e.g., amino acids, monoamines) is mediated, which would then provide insight into how they regulate various physiological events.

The seastar neural network is similar to other echinoderms, comprising a circumoral nerve ring located at the oral region connected to a radial nerve cord (RNC) which runs centrally along the length of each arm to the distal eye spot (9). Electrochemical signal transduction between neurons allows an organism to react to various internal and external stimuli which regulate their behavioral and physiological changes (10). Nerves produce and secrete chemicals, including small molecule neurotransmitters, in response to internal or external stimuli to regulate the animal's behavior (11, 12). Neurotransmitters are released from the neural synaptic vesicles into the synaptic cleft, where they are received by neurotransmitter receptors on target cells. While the neuroanatomy and neurotransmitter peptides (neuropeptides) from some seastar species have been extensively investigated (12–16), less is known regarding the small molecule neurotransmitters and their biosynthetic pathways.

The four major groups of small molecule neurotransmitters are acetylcholine, monoamines, soluble gases and amino acids (17). Additionally, the four monoamine neurotransmitters are divided into two subsets on the basis of their chemical structure: the catecholamines, that include dopamine, norepinephrine, and epinephrine, and the indolamines, that include serotonin (18). In echinoderms, neurotransmitters have been investigated within the areas of locomotion, metamorphosis (19–22), arm autotomy (23, 24), and oocyte maturation (25, 26). The roles that small molecule neurotransmitters play in regulating adult growth and reproductive processes is less well studied, which is likely due to the increased complexity of the associated pathways of bioactivity.

Here, we report on the use of a non-targeted metabolomics approach to comparatively assess the presence and distribution of small molecule neurotransmitters within the COTS RNCs. We show that neurotransmitter abundance can vary significantly between males and females, and in animals of differing satiation state. We further annotate the genes involved in neurotransmitter biosynthesis pathways, as well as demonstrate the spatial localization within the RNC for serotonin, GABA, and histamine.

## Materials and methods

### Animals and radial nerve cord tissue collection

Adult COTS *(Acanthaster planci)* were collected from the central sector of the GBR, Australia, in the early autumn months (March- April; non-reproductive) and transported to the Australian Institute of Marine Science (AIMS), Townsville, where they were kept in isolation tanks until needed. RNC tissues were dissected from ~5 amputated arms of each individual (as per Smith et al 2017) (14) and separated into four experimental groups: (1) male control (MC), (2) female control (FC), (3) male food-deprived (MFD) and (4) female food-deprived (FFD). Gender was established as described by Smith et al. (14). For food-deprivation, COTS were kept without food for ~90 days. Approximately 0.20 g of RNC tissue was collected from each individual COTS and immediately stored at −80°C.

### Radial nerve cord sample preparation for mass spectrometry

RNC analysis was performed in triplicate for each of the four experimental groups. Tissues were processed for metabolomics using methods described previously (27). In brief, the weight of each RNC was recorded then tissue was dissolved in 1.5 ml of cold methanol:MilliQ water (MeOH:H_2_O, 1:1, Millipore, Bedford, MA, USA) and homogenized with a TissueRuptor (25 Hz, 5 min cycle; Qiagen, US). Samples were centrifuged at 16,000 xg for 10 min. Finally, supernatant was collected, freeze-dried, and kept at −80°C. Freeze-dried samples were re-suspended to 15% of the original volume by adding 30 μl MeOH then diluted with 120 μl of H_2_O to produce a 20:80 MeOH:H_2_O solution. The extract sample was stored at −80°C until use.

### Metabolite profiling with high-resolution accurate mass spectrometry (HRAMS)—UHPLC-QToF-MS analysis

Samples were analyzed using an Agilent UHPLC-QToF-MS system (Agilent Technologies, Santa Clara, CA, USA) comprising a 1290 UHPLC coupled to a 6520 Accurate-Mass Quadrupole Time-of-Flight Mass Spectrometer (QToF-MS, Agilent Technologies, Santa Clara, CA, USA) in both positive and negative mode from *m/z* 100 to 1700 for all samples at a scan rate of 0.8 cycles/s. Instrument resolution was 9,000–11,700 across the data acquisition range. The instrument was calibrated in the extended dynamic range with the Agilent ESI-TOF Reference Mass Solution Kit. LC-MS set up parameters were as described previously (27, 28). Chromatographic separation was achieved using a Phenomenex Gemini-NX C18 HPLC column (150 × 2.0 mm, 3 μm, Phenomenex, Lane Cove, NSW, Australia). The mobile phase consisted of (A) 0.1% TFA (Sigma-Aldrich, Sydney, Australia) in H_2_O and (B) 0.1% TFA in acetonitrile (LabScan Analytical Science, Taren Point, Australia). The elution gradient increased from 80% A: 20% B to 0% A: 100% B over 25 min, followed by a 2 min hold, then returned to starting conditions over 2 min followed by a 6 min re-equilibration before the next injection. A sample volume of 20 μl was injected for each UHPLC run. Blanks and pooled quality control samples were intercalated throughout the sample series to control for any acquisition-dependent variation. The samples and standards were filtered using a 0.2 μm PTFE membrane filter (Phenomenex, Torrance, CA, USA) before analysis. Continuous internal calibration [Agilent ESI-TOF Reference Mass Solution Kit: internal lock masses *m/z* 121.0509 (protonated purine) and 922.0098 (protonated hexakis)] was performed in parallel with sample analyses.

### Data processing, molecular formula generation, chemometric analyses and identification of compounds

Data analysis was performed using Agilent MassHunter Qualitative software (Version B.05.00). The Molecular Feature Extractor algorithm within MassHunter Qualitative analysis software was used to extract chemically qualified molecular features from the UHPLC-QToF-MS data. Data processing and formula generation details are described in Bose et al, 2016 (27). Samples from the different experimental groups were evaluated separately by multivariate analysis. Feature-extracted sample files were transferred into Agilent GeneSpring software version 12.0 (Agilent Technologies, Santa Clara, CA, USA) for alignment and to compile a data matrix.

The Total Ion Chromatograms (TICs; mass range of 100 to 1500 *m/z* at a retention time window 2–24 min) were visually compared. All compounds in this range were extracted (Extracted Ion Chromatograms) and used to build the data matrix for subsequent multivariate analysis. The intensity of each *m/z* signal was normalized against the two internal lock masses after which the Find by Molecular Feature algorithm in Agilent Mass Hunter was applied; this algorithm identifies the compound(s) based on the absolute measured abundance of each *m/z* found to be persistent across proximal retention times, then calculates the relative abundance of each compound peak (i.e., integrate). For more information relating to these methods see (27). Exact tissue mass from each sample was then used to normalize the relative abundance data proffering semi-quantitative data.

The data matrix (positive mode: 488 variables, 12 observations; negative mode: 205 variables, three observations from MFD omitted from the analysis due to unsuccessful extraction/acquisition) was imported into SIMCA-P+ version 13.0 (MKS Umetrics AB, Umeå, Sweden). Data were pre-treated by log10 transformation and mean centring prior to analysis. Data were also analyzed using pareto and unit variance scaling but such scaling was deemed superfluous for feature selection in this dataset and thus only mean centred analysis/results are shown. The data matrix was analyzed using principal component analysis (PCA) and orthogonal projection to latent structures-discriminant analysis (OPLS-DA). An unsupervised analysis by PCA identified outliers and determined any groupings or trends (12 samples, including three biological replicates). For OPLS-DA models a minimum threshold of Variable Importance in Projection (VIP); VIP>1 was used for variable selection (= “importance”) (28). The *m/z* values, collated from the molecular feature extraction and molecular formula generation of the metabolic profiles, were used to interrogate the METLIN, HMDB and in-house databases and compounds identified based on a match to accurate monoisotopic mass. Compound identification details are given in (24, 25). GraphPad Prism software was used to draw the box plots (GraphPad Software, San Diego, CA). The stability and reproducibility of data was ensured by the quality control samples measured during the data acquisition time (29).

### Biosynthesis pathway annotation

*Homo sapiens* and invertebrate neurotransmitter biosynthetic pathway enzyme genes were obtained from the National Centre for Biotechnology Information (NCBI; www.ncbi.nlm.nih.gov), and then used as queries for BLASTp searches of published COTS genome and transcriptome databases (8). BLASTp searches were performed using the CLC Main Workbench Version 6.0 with an e-value cut-off 10^−3^. SMART (a simple modular architecture research tool) was used to identify and annotate conserved domains present in individual proteins (30). Illustrator for Biological Sequences (IBS, Version 1.0) was used to draw schematic diagrams for protein domains (31).

### L-glutamic acid spawning inhibition assay

Gravid adult female *A. planci* were collected in the breeding season (July) from the reef of Okinawa, Japan. Modified Vant' Hoff's artificial seawater (ASW) adjusted to pH 8.2 with 0.02 M borate buffer was prepared (32), and the ovaries of mature female were excised and cut using scissors into small fragments containing only a few lobes. Ovarian fragments were incubated at 25°C in ASW containing 1-methyladenine (1-MeAde) at 1 μM in the presence of L-glutamic acid (Sigma-Aldrich, St. Louis, USA), at 0.4–100 μM. After 60 min incubation, ovulation activity was determined as follows: (+++) Ovulation occurred completely; (++) ovulation occurred, but less than half oocytes remained within the ovary; (+) ovulation occurred partially; and (–) no ovulation occurred. The scores were converted to numerical values (+++ = 100; ++ = 67; + = 33; - = 0) so that the effective dose for inducing gamete spawning in 50% of ovarian fragments could be determined graphically. The mean was determined from four independent samples and standard error of the mean (SEM).

### Histology and immunocytochemistry of COTS radial nerve cord

RNC were dissected from adult COTS (isolated male and female controls) and fixed in 4% paraformaldehyde solution at 4°C overnight before embedding in paraffin wax and sectioning at 8 μm using a Leica microtome. Sections were mounted on slides and deparaffinised using xylene-ethanol (EtOH) washes. Tissues for histology were prepared by immersing in Harris's Hematoxylin stain for 10 min then rinsed in running water for 10 min followed by 95% EtOH. Tissue was then dipped in Eosin stain (1:1 dilution) for 10 min and rinsed with 95% EtOH for 5 min followed by two 5 min rinses with 100% EtOH. Slides were mounted using DPX media and viewed using a Leica DM550 microscope with Leica digital camera to capture images.

For immunocytochemistry, sections (8 μm) were blocked using 4% goat serum (Sigma) in phosphate buffered saline with Tween 20 (PBST) and incubated at room temperature for 1 h. Primary antibodies (anti-rabbit GABA, anti-rabbit histamine, and anti-rat serotonin; all ImmunoStar) were prepared at 1:500 dilution in PBST and incubated with sections overnight at 4°C. The anti-rabbit GABA and anti-rabbit histamine were used as a positive controls as they have been confirmed in other echinoderms (11). Tissues were washed three times with PBST for 5 min. Secondary antibodies (anti-rat Alexa Fluor® 568 and anti-rabbit Alexa Fluor® 488 at 1:200 in PBST) were applied to sections for 1 h at room temperature in the dark. DAPI nuclear stain (300 nM) was added and incubated for 10 min. Negative controls included no secondary antibody or preabsorbed anti-serotonin (with serotonin (1 mM) in PBST, incubated overnight at 4°C). Tissues were washed three times for 5 min with PBST, and mounted using Vectashield mounting medium (VECTASHIELD®). Slides were viewed under fluorescence using a Leica DM550 microscope with Leica digital camera.

## Results and discussion

### Global metabolite profiling of crown-of-thorns seastar radial nerve cord

**Figure 1:**
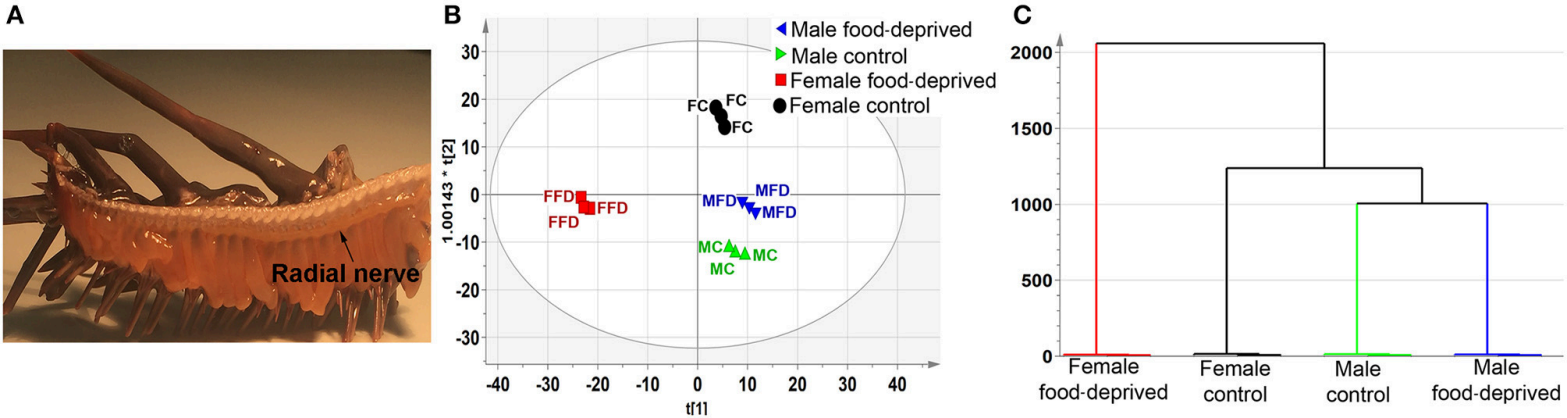
Untargeted metabolomics analysis of Crown-of-Thorns Seastar radial nerve cord (RNC). **(A)** Whole-mount micrograph showing location of the COTS RNC. **(B)** OPLS-DA scores plot, predictive component, t(1), versus orthogonal component, t0(1), showing the supervised separation between the four sample classes. The ellipse represents the Hotelling's T2 95% confidence interval for the multivariate data. **(C)** Dendrogram displaying the results of the Hierarchical Clustering Analysis (HCA). The plot shows the number of clusters as a function of the vertical coordinate.

LC-MS-based neurotransmitter profiling can now be routinely performed on tissues and biological fluids (33). The identification and measurement of metabolites can provide an excellent insight into the biochemistry of tissue function and inform upon key targets for further analysis, including small molecule neurotransmitters. In our study, RNC tissues were dissected from the amputated arms of male and female COTS (Figure 1A), and following a long period (~90 days) of food deprivation, then analyzed by LC-MS-based metabolite profiling (see Figure S1 for LC chromatograms). Interrogation of the PCA and OPLS-DA loadings plot with VIP identified numerous metabolites based on multivariate analysis (Table S1). To retrieve the variables information related to sample classification, a supervised OPLS-DA method was used. The OPLS-DA scores (Figure 1B) shows a complete separation in the predictive (horizontal) component, with R2X (cumulative; percentage of all response variables explained by the model) = 7.3%, R2Y (cumulative; the percentage of all observation or sample variables explained by the model) = 99.2% and Q2 (cumulative; the percentage of all observation or sample variables predicted by the model) = 79.1%, simplifying interpretation of the complex dataset by establishing the variables that contribute to the differences in the four experimental groups based upon their overall chemical profiles. Variation observed in the orthogonal (vertical) component is unrelated to sample class differences but may warrant further investigation given that these are biological replicates. Clustering analysis, based on the metabolites present in the RNC, shows that unique sets of compounds are present in female food-deprived (FFD) samples in comparison to other sample groups; the first two scores vectors [t (1) versus t (2)] are plotted in Figure S2. The scores plot also confirmed that no technical outliers were present. The variables responsible for any groupings or clusters in the data could be determined from the loadings plot. Overall, the male and female control (MC and FC) COTS RNC contained a greater number of ionisable metabolites than individuals that were food-deprived, which suggests an increased metabolic activity in satiated animals. The hierarchical clustering analysis dendrogram demonstrates that the FFD group has the highest level of dissimilarity (Figure 1C).

### Targeted analysis of metabolites in male and female COTS radial nerve cord

The global metabolite data provided an excellent resource to explore specific metabolite differences in COTS RNC, including between gender and after food-deprivation. The focus was on small molecule neurotransmitters, i.e., low molecular weight amino acids and lipid-derived prostaglandins (PGs), which have previously been implicated in animal reproduction and growth, and for which their biosynthesis pathways have been established in other phyla (34–36).

### GABA, histamine, and serotonin

**Figure 2:**
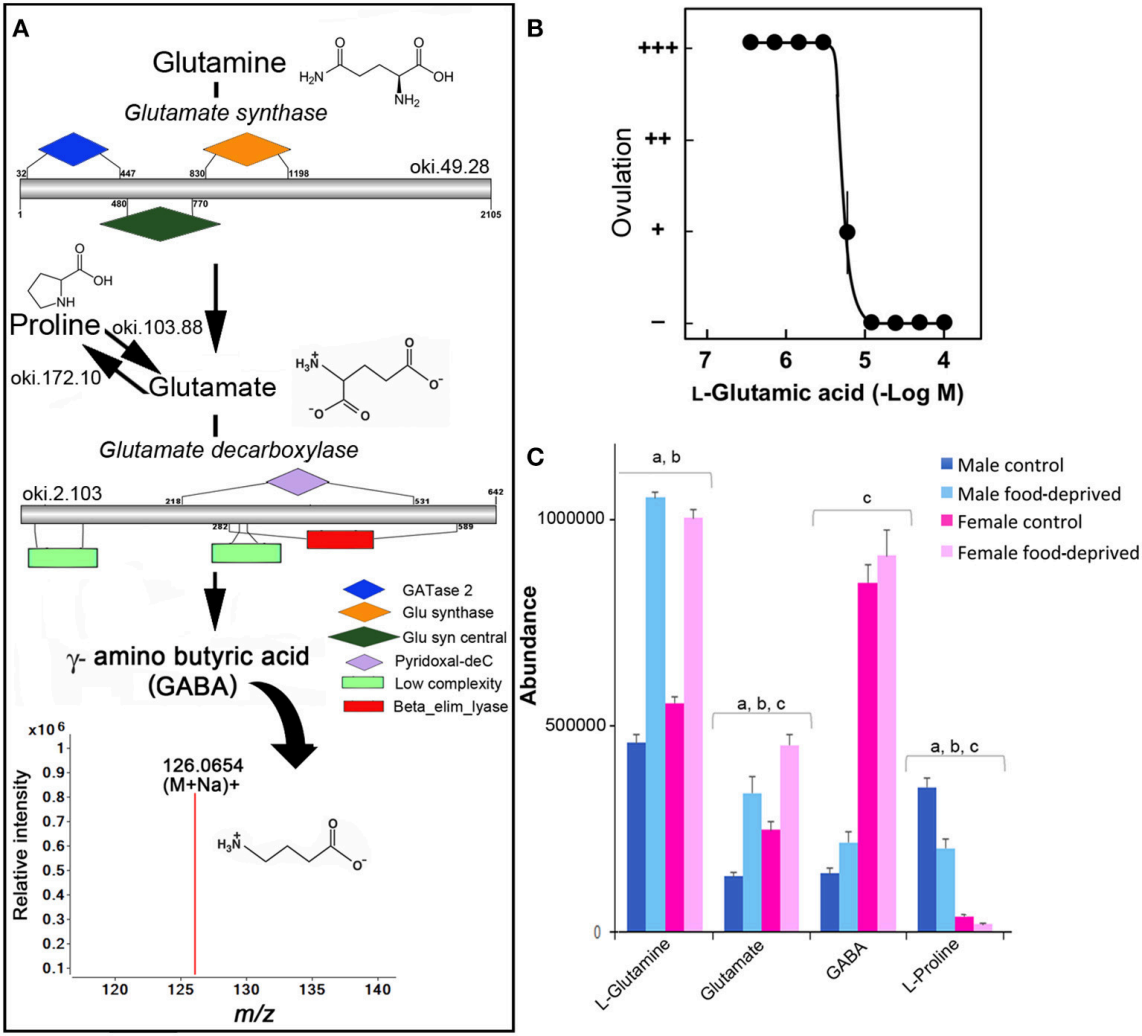
Identification of GABA in the Crown-of-Thorns Seastar. **(A)** Schematic showing GABA biosynthetic pathway, including enzyme structural organization, domains and mass spectrum for GABA (*m/z* 126.0654). See File S1 for gene ID and protein sequences for enzymes. **(B)** Effect of L-glutamic acid on 1-MeAde induced spawning in Crown-of-Thorns Seastar ovarian fragments. (+ + +) Ovulation occurred completely; (++) ovulation occurred, but less than half oocytes remained within the ovary; (+) ovulation occurred partially; and (–) no ovulation occurred. Symbols and bars represent the mean for four independent samples and SEM, respectively. **(C)** Graph showing tentative identification and abundance (mean + SD) of GABA and its derivatives in the radial nerve cord. Significant differences of *p* < 0.05 are denoted as - a, between control and food-deprived in male; b, between control and food-deprived in female; and c, between male and female COTS. Abundance = the total volume (*m/z* x retention time x abundance) of the ions associated with this compound.

GABA has been found in the RNC, tube feet and digestive system of adult *Asterias rubens* (37, 38), and is associated with pharynx protractor muscle, podia and tube foot contraction (39, 40). A search for homolog enzymes involved in the biosynthesis of GABA utilizing the COTS genome (8) and BLASTp analysis identified all homolog enzymes for GABA biosynthesis from glutamine (Figure 2A). The detection of ions consistent with GABA (*m/z* 126.0654 at R _*T*_ 8.22 min), which likely acts through receptors that have been previously annotated from the COTS genome (8), supports this. To our knowledge, studies of glutamate or GABA in echinoderm reproductive biology is limited, however, L-glutamic acid (the pronated form of glutamate) has been shown to act as a spawning inhibitor in seastars *Patiria pectinifera* (41). The effect of L-glutamic acid on COTS was tested *ex-vivo* on ovarian fragments (Figure 2B) and was found to inhibit ovulation, despite the presence of 1-MeAde; 1-MeAde is a maturation-inducing hormone that resumes meiosis of seastar oocytes. The concentration required for 50% inhibition was 5.6 ± 0.6 μM L-glutamic acid. This demonstration further corroborates the previous study, confirming L-glutamic acid inhibits ovulation in seastars, and identified the compound and the GABA pathway as a potential target for COTS biocontrol. Although speculative, the significant increase of L-glutamate following food deprivation may provide some insight toward a causation for reduced populations of COTS by means of reproductive inhibition.

GABA has a recognized role in regulating the vertebrate gonadotropin-releasing hormone (GnRH) neurons where it is primarily inhibitory (36). A GnRH-like peptide was recently discovered in COTS (42), although its function and regulation is still unclear. In control COTS, L-glutamine, glutamate, and GABA are more abundant in FC than MC (Figure 2C). Interestingly, L-proline, which can also be catabolised to glutamate (Figure 2A), was found to be less abundant in FC compared to MC, possibly indicating its ultimate conversion via glutamate to GABA in FC. Following food deprivation, only a small increase in GABA was observed (Figure 2C). Despite the lack of general knowledge in the area of echinoderm small molecule neurotransmitters following food deprivation, there is some information from vertebrate studies. For example, in the rat brain, long-term food deprivation induces an increase in GABA (43, 44). For GABA, there is a reduction in the upstream turnover of GABA metabolizing pathways (i.e., that results in a decrease in GABA synthesis).

**Figure 3:**
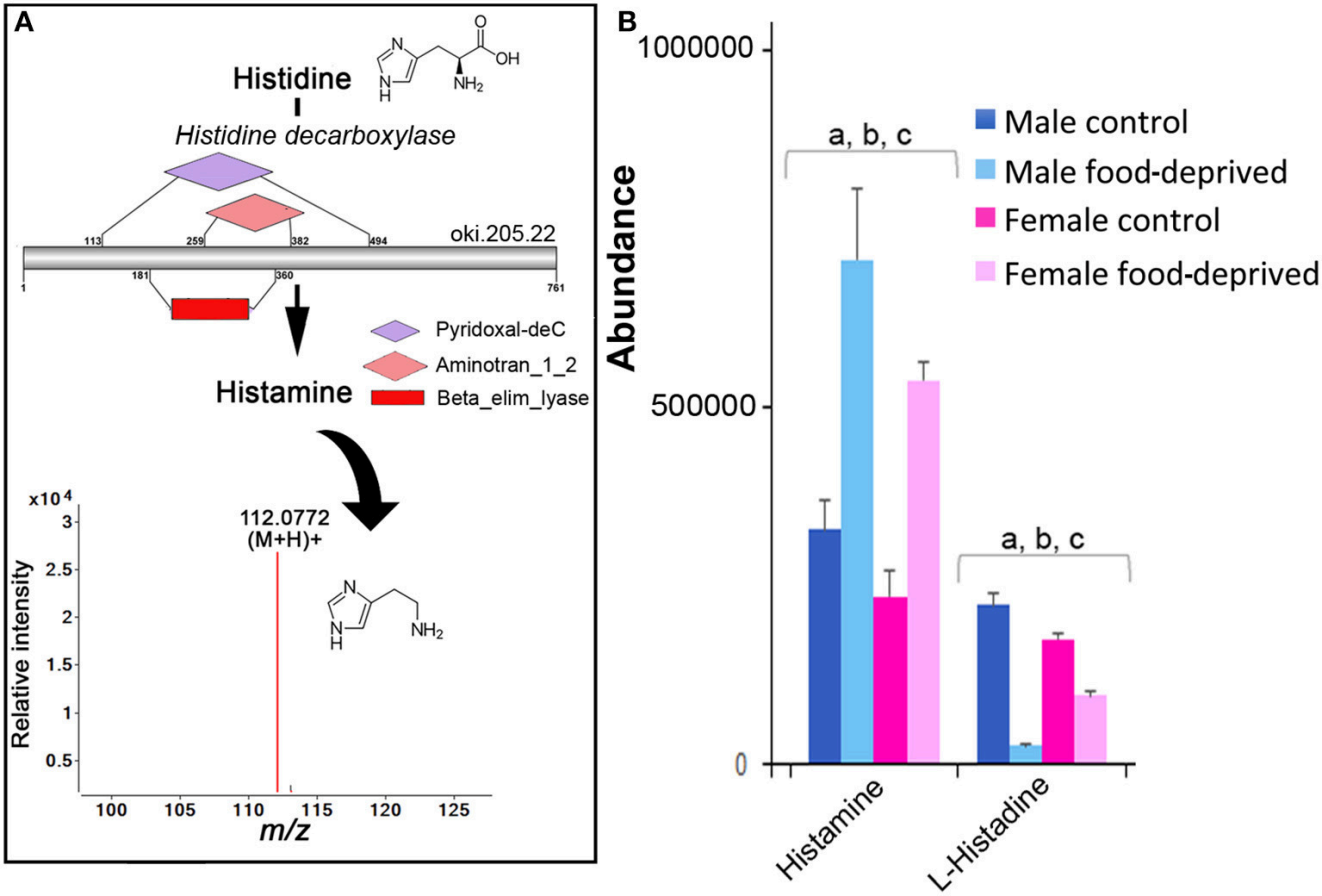
Identification of histamine in the Crown-of-Thorns Seastar. **(A)** Schematic showing histamine biosynthetic pathway, including enzyme structural organization, domains and mass spectrum for histamine (*m/z* 112.0772). See File S1 for gene ID and protein sequences for enzymes. **(B)** Graph showing tentative identification and abundance (mean + SD) of histamine from histidine in the radial nerve cord. Significant differences of *p* < 0.05 are denoted as—a, between control and food-deprived in male; b, between control and food-deprived in female; and c, between male and female COTS. Abundance = the total volume (*m/z* x retention time x abundance) of the ions associated with this compound.

There has not yet been any comprehensive analysis of histamine in adult echinoderms, although histamine has been found in the sea cucumber radial nerve cord, tentacles, and papillae of the body wall (11) and several tissues of the “slender armed” seastar, *Luidia clathrata*. The knowledge surrounding the role of histamine in echinoderms, to date, is restricted primarily to its involvement in fertilization, metamorphosis, and settlement in echinoid species (e.g., sea urchin) (21), and it may be involved in asteroid toxicity (45). As shown in Figure 3A, we identified histidine decarboxylase in the COTS genome, an enzyme required for histamine biosynthesis from its biosynthetic precursor, histidine. Ions consistent with histamine (*m/z* 112.0772 at R _*T*_ 8.80 min) were detected. Most interestingly, histamine and histidine are more abundant in the MC (Figure 3B). This finding is consistent with the discovery of elevated levels of histidine decarboxylase in testes of sea urchins, and the corresponding low/absent levels in ovaries and eggs (46). Following food-deprivation, there is a significant increase in COTS RNC histamine (Figure 3B). In the rat brain, long-term food deprivation also induces an increase in histamine, which may be due to changes in homeostatic control of energy that is modulated by histamine (43).

**Figure 4:**
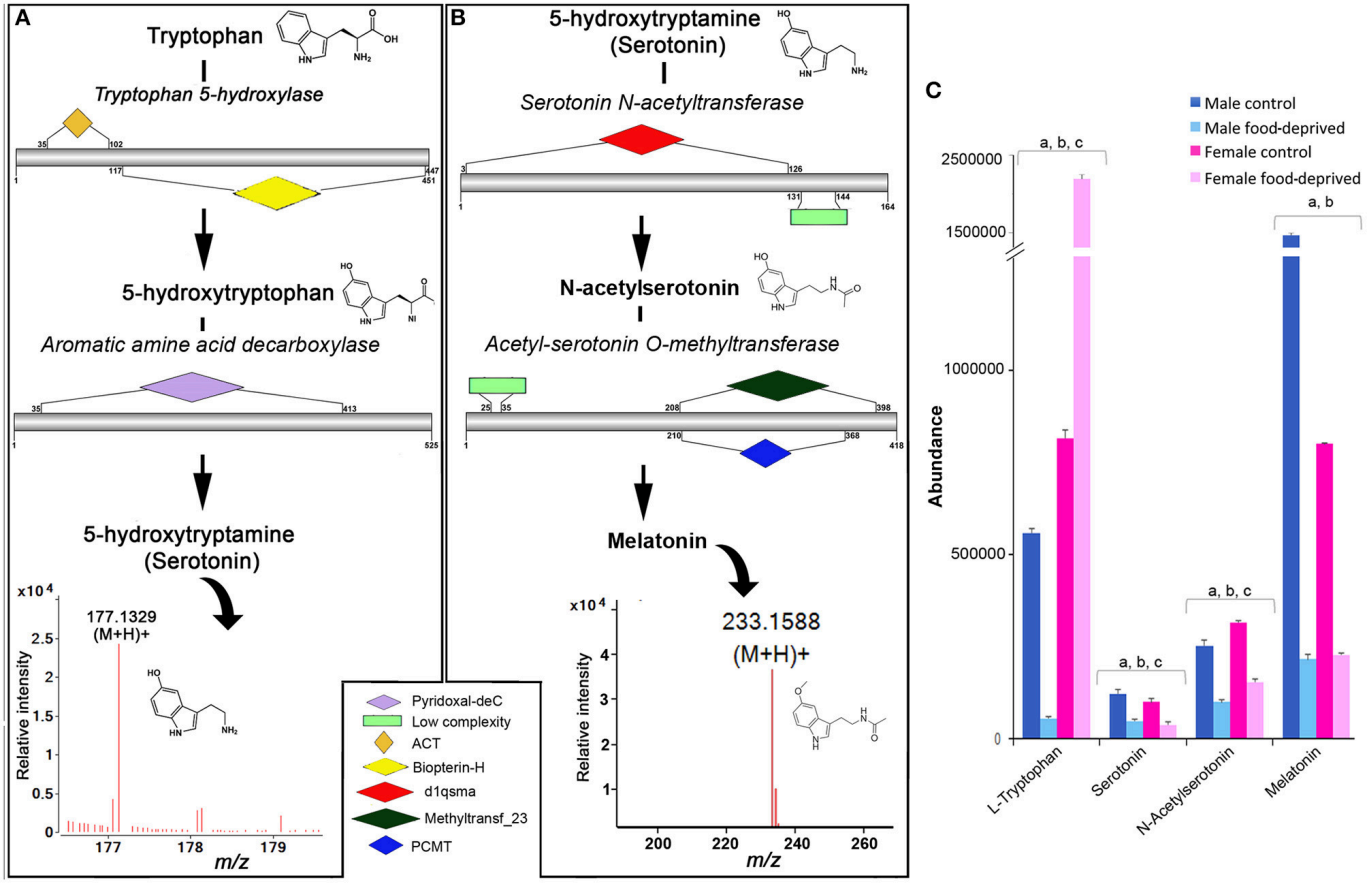
Identification of serotonin and melatonin in the Crown-of-Thorns Seastar. Schematic showing **(A)** serotonin and **(B)** melatonin biosynthetic pathways, including enzyme structural organization, domains and mass spectrum for serotonin (*m/z* 177.1329) and melatonin (*m/z* 233.1588). See File S1 for gene ID and protein sequences for enzymes. **(C)** Graph showing tentative identification and abundance (mean + SD) of serotonin, melatonin and their derivatives in the radial nerve cord. Significant differences of *p* < 0.05 are denoted as—a, between control and food-deprived in male; b, between control and food-deprived in female; and c, between male and female COTS. Abundance = the total volume (*m/z* x retention time x abundance) of the ions associated with this compound.

In echinoderms, serotonin has been found to play a role in metamorphosis (19, 22), oocyte maturation (26), feeding during larval settlement (47), and arm regeneration (48). We identified all enzymes within the COTS genome required for the biosynthesis of serotonin from its biosynthetic precursor, tryptophan (Figure 4A) and for melatonin, which is derived from serotonin (Figure 4B; see File S1 for protein sequences). Ions consistent with serotonin (*m/z* 177.1329 at R_T_ 6.85 min) and melatonin (*m/z* 233.1588 at R_T_ 9.1 min) detected by UPHPLC-MS. No significant difference was observed in serotonin abundance between genders, however, there was a significant decrease in serotonin following food deprivation, for both FFD and MFD (Figure 4C). In the rat brain, long-term food deprivation induces a down-regulation of serotonin transporters (43). The decrease in serotonin in food-deprived COTS contrasts the trend observed for tryptophan, which significantly decreases in MFD but increases significantly in FFD.

For melatonin, we found that the MC had significantly higher levels than FC, while both males and females had significantly decreased melatonin levels following food deprivation. Although speculative, it is possible that under the stress of food deprivation, inclusive of diminished essential amino acid dietary uptake, the downstream biosynthetic pathways of tryptophan are altered in response to the reduced metabolic demands associated with growth and reproduction (49–51). In a metabolomics study of the freshwater invertebrate, *Daphnia magna*, exposed to dietary and environmental stresses, levels of essential free amino acids such as tryptophan increased when animals were food deprived (51). Although speculatively, we propose that the role tryptophan plays in fertilization biology as a chemical attractant between sperm and egg may be influenced by this environmental stress and as such should be considered for further analysis.

In context of COTS behavioral biology, it is often observed that juveniles and smaller adults prefer nocturnal feeding whilst the bigger animals are seen to be diurnal feeders (52). Melatonin, the downstream derivative of serotonin is the key regulator for seastar circadian rhythm which regulates their nocturnal exhibition (53). Melatonin levels are found to decrease in response to this apparent down regulation of the tryptophan—serotonin metabolomic pathway, which may unveil changes to the feeding behavior of COTS, and therefore should be considered for future behavioral studies.

**Figure 5:**
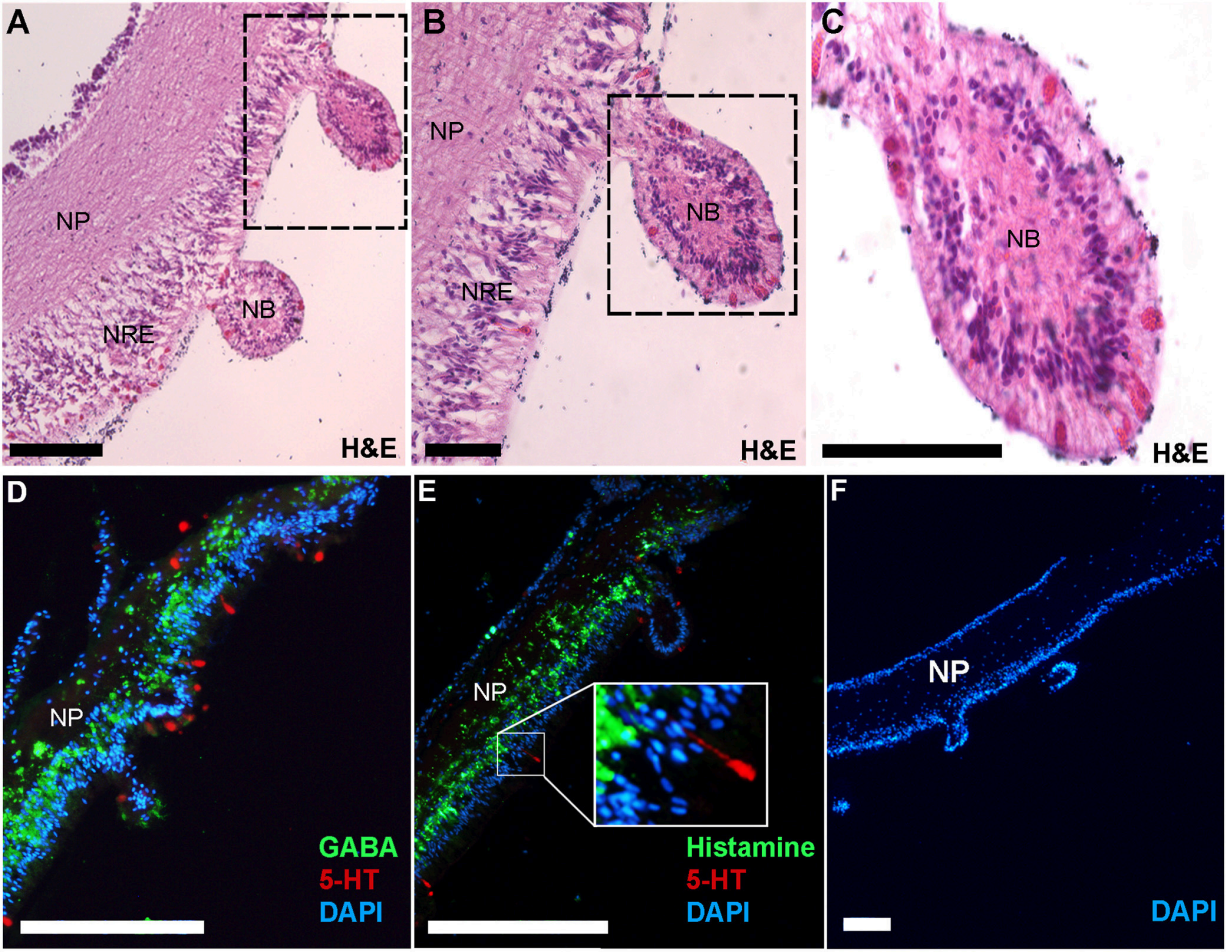
Histological analysis and immunofluorescent localization of neurotransmitters in the Crown-of-Thorns Seastar radial nerve cord. **(A–C**) Transverse section of adult COTS RNC tissue stained with hematoxylin and eosin (H&E). Dashed box shows region of magnification. NP, neural plexus; NRE, nuclear region epithelium; NB, neural bulb. Scale bars = 20 μm. (**D, E**) Immunofluorescence analysis using anti-serotonin (5-HT), anti-GABA, and anti-histamine. **E**, inset shows magnification of 5-HT immunoreactive cell. **(F)** Negative control using no primary antibody. DAPI nuclear stain (blue) was used as a nuclear counterstain. Scale bars = 100 μm.

Immunofluorescent localization was used to elucidate the spatial distribution of GABA, histamine and serotonin in the COTS RNC. First, RNC isolation followed by histological examination allowed for the visualization of the RNC structure, showing regions of the neuropil and neural epithelium (Figures 5A–C) in the neural bulb projections extending along the ectoneural surface, which to our knowledge, have not yet been described as a structure in echinoderm neural systems. Serotonin localized to regions along the ectoneural surface, notably on the nuclei dense areas of neural bulbs (Figures 5D, E). GABA and histamine were observed within the neuropil fibers (Figures 5D, E), although GABA was more prominent within the nuclear region epithelium of the ectoneural surface with some indication that it is present within the perikaryal. In *A. rubens*, GABA is confined to the ectoneural region of the RNC (37), whereas it can be seen sparsely distributed in the hyponeural neuropil of the COTS. Histamine was observed most densely within nuclear region epithelium of the ectoneural surface in the perikaryal and along neural fibers (Figure 5E). The negative control showed no staining (Figure 5F) with antibody controls in Figure S3. The presence of GABA in COTS RNC tissue further implicates GABAergic neurons involvement in the seastar RNC (37) and should be considered for future studies, particularly relating to COTS reproductive and feeding biology.

### Dopamine, epinephrine, and octopamine

**Figure 6:**
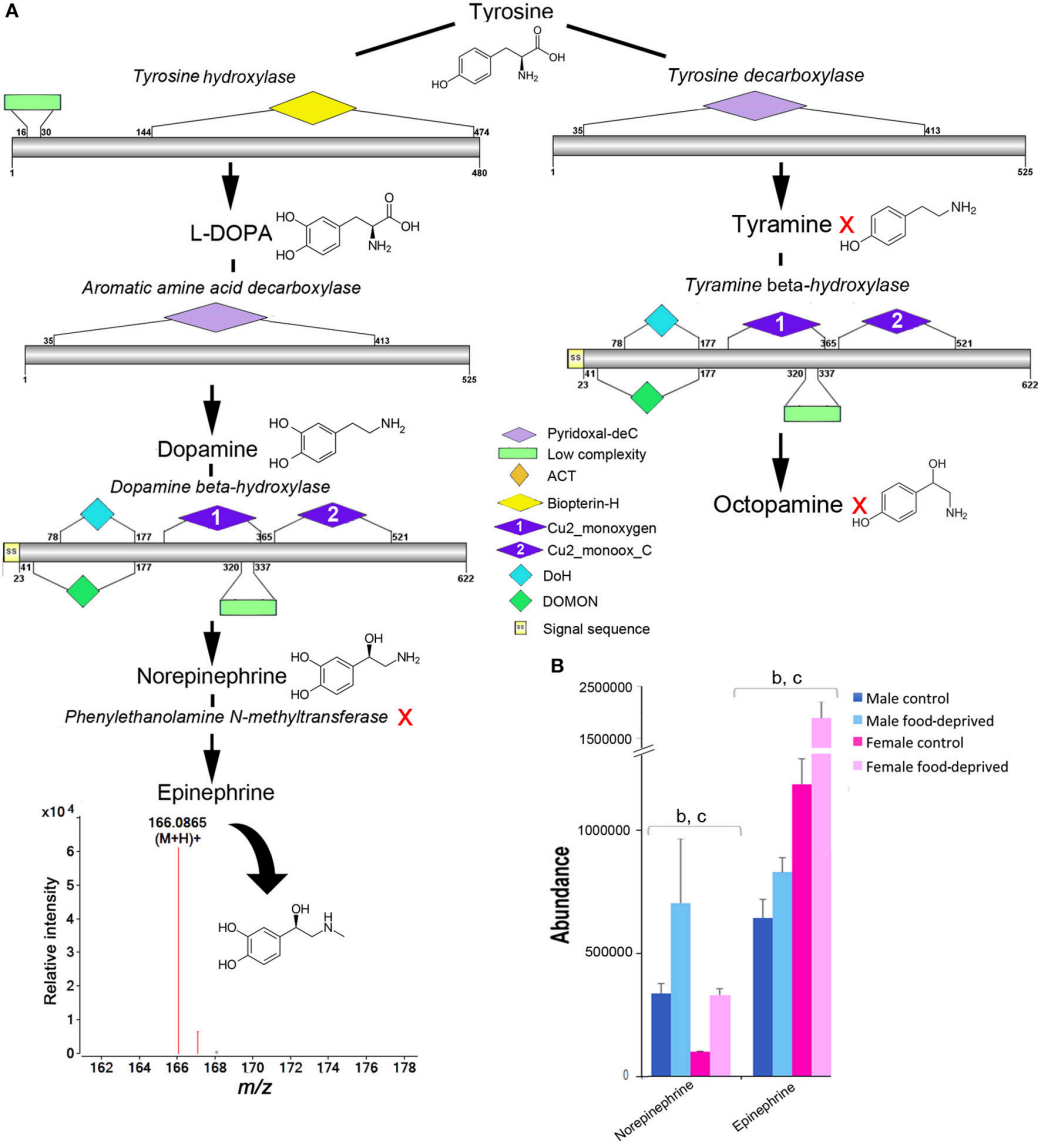
Identification of epinephrine and octopamine in the Crown-of-Thorns Seastar. **(A)** Schematic showing biosynthetic pathway, including enzyme structural organization, domains and mass spectrum for epinephrine (*m/z* 166.0865). A red X indicates the component was not identified. See File S1 for gene ID and protein sequences for enzymes. **(B)** Graph showing tentative identification and abundance (mean + SD) of norepinephrine and epinephrine in the radial nerve cord. Significant differences of p<0.05 are denoted as - b, between control and food-deprived in female; and c, between male and female COTS. Abundance = the total volume (*m/z* x retention time x abundance) of the ions associated with this compound.

Tyrosine is a common precursor for the biosynthesis of dopamine, epinephrine and octopamine. All enzymes required for the biosynthesis of dopamine, norepinephrine and octopamine were identified within the COTS genome (see File S1 for protein sequences), with the exception of phenylethanolamine N-methyltransferase; this enzyme is known to modify norepinephrine into epinephrine (Figure 6A). Despite this, our metabolite analysis found that ions consistent with epinephrine were present (*m/z* 166.0865 at R_T_ 6.95 min). It was most interesting to observe that norepinephrine (also known as noradrenaline) and epinephrine (adrenaline) levels were significantly higher in food-deprived COTS (Figure 6B). Also, it was surprising that despite containing the biosynthetic enzymes for tyramine and octopamine synthesis, neither of these metabolites were detected. A low concentration of tyramine has previously been detected in the radial nerve tissue of the sunflower seastar, *Pycnopodia*, radial nerve tissue (54).

As deuterostome invertebrates, echinoderms have always held a curious position with regards to neurotransmitter profiling. There is evidence that molecules traditionally reserved as being either invertebrate only (i.e., octopamine) or vertebrate only (i.e., norepinephrine) have been found in the echinoderm radial nerve (55), although there are higher levels of norepinephrine than octopamine (56). In the current study, we assume that the levels of tyramine and octopamine were too low for detection, or that they are rapidly metabolized during our tissue processing method. In echinoderms, the existence of octopamine is supported by a bioassay showing octopamine-induced tonic contraction in sea urchin spine muscle (57), while norepinephrine/noradrenaline has been shown by histofluorescence to increase during seastar arm tip regeneration (58). In other invertebrates, including *Drosophila* and *Anopheles*, the metabolic influence of octopamine has been well established, indicating a critical role in regulating the animal's resistance to starvation, as well as being essential for oviposition and hatching rates (59, 60). Tyramine, however, is shown to have opposing effects on octopamine and acts antagonistically (59). Given that octopamine is generally found in high abundance in invertebrates, it was interesting that we could not identify it in our metabolite data, although this is consistent with other echinoderm studies where only low levels of octopamine were reported (61). Further investigation is required to clarify the coexistence of norepinephrine/noradrenaline and octopamine in COTS.

Similarly, acetylcholine (Ach), which has been described as a main transmitter of echinoderm motor systems (62, 63), was absent from our metabolite data. A targeted analysis has previously identified Ach within isolated RNC and podia of *A. rubens* (64), where it has been shown to be an excitatory neurotransmitter (40, 65, 66). In our study, we speculate that the high molecular turnover of Ach, which could be further exacerbated during the process of tissue preparation and isolation (67), may have contributed to Ach degradation prior to detection. In support of this, it is known that Ach localization from echinoderm RNC cholinergic neurons (nerve cells that contain Ach) is challenging, so its presence is typically reliant on the detection of Ach-degrading enzyme, acetylcholinesterase (64). The annotated COTS genome revealed a choline acetyltransferase-like (oki.175.31; converts choline to Ach) gene and at least three acetylcholinesterase-like isoform genes (oki.77.2, 77.83, 77.84), all of which have high expression in the radial nerve cord and podia (8).

### Prostaglandin (PGs)

**Figure 7:**
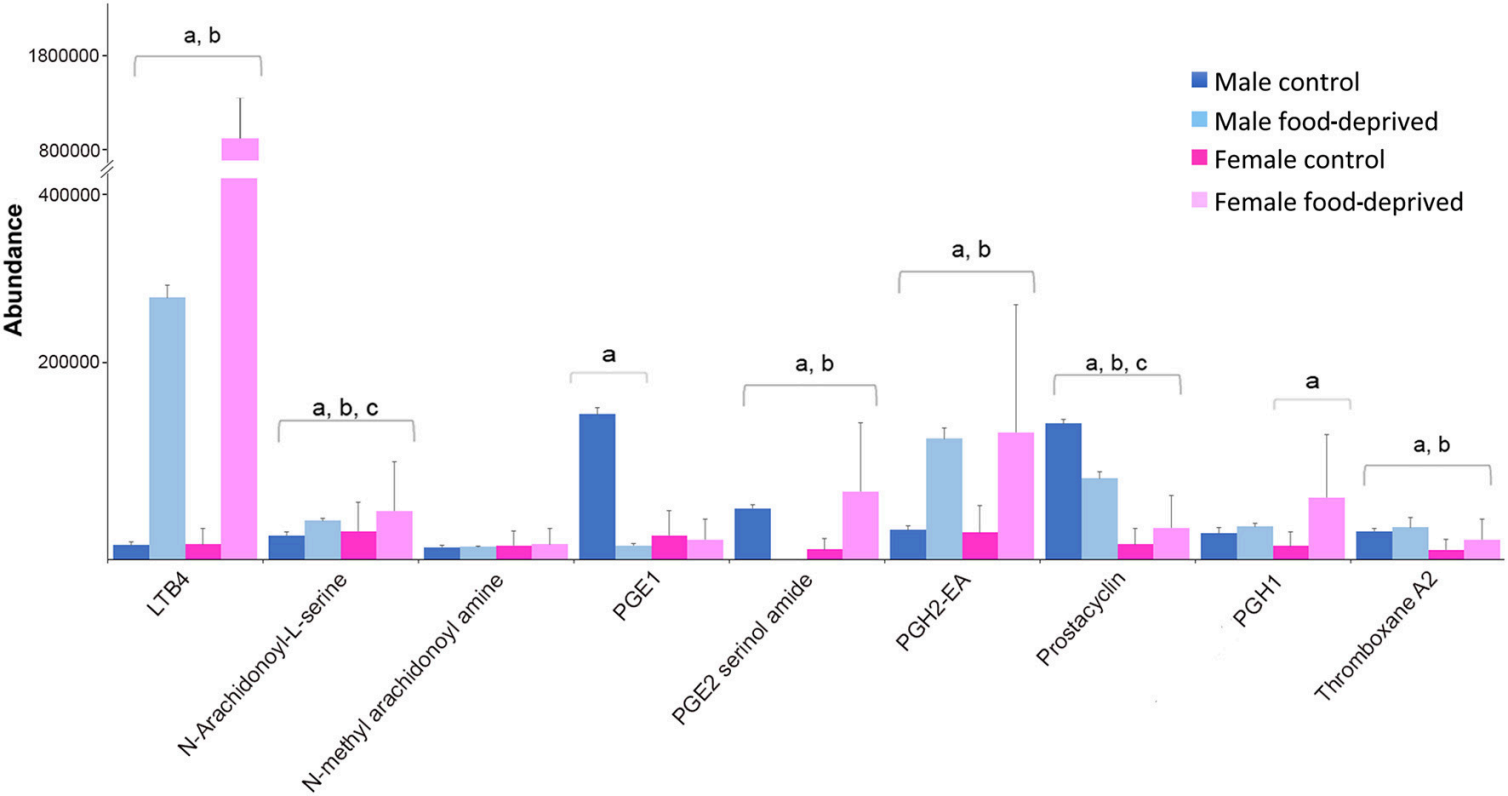
Graph showing tentative identification and abundance (mean + SD) of prostaglandins and their derivatives in the Crown-of-Thorns Seastar radial nerve cord. Significant differences of *p* < 0.05 are denoted as—a, between control and food-deprived in male; b, between control and food-deprived in female; and c, between male and female COTS. Abundance = the total volume (*m/z* x retention time x abundance) of the ions associated with this compound.

PGs are lipid autacoids derived from arachidonic acid that along with other biologically active derivatives of polyunsaturated fatty acids have been detected in a large number of invertebrates (68). Invertebrates use PGs to control gametogenesis, ion transport and defense [reviewed by (69)]. In seastars, various PGs have been implicated in the induction of the complete maturation program, including maturation by germinal vesicle breakdown, followed by fertilization and development into larvae (70). Figure 7 shows the abundance of PGs identified in male and female COTS, and during food deprivation. The observed lower levels of PGs in FC compared to MC COTS may be related to the ongoing reproductive processes including oocyte development. When food-deprived, these processes may slow or cease, and valuable resources are re-directed elsewhere to ensure survival. Overall, levels of leukotriene B4, PGE2 serinol amide, PGE2 EA, PGH1 and thromboxane A2, were increased after food deprivation. These metabolites are all indicators of food intake (71–74), and as for arachidonic acid from dietary fatty acids, they are known to have roles in echinoderm reproduction and are accumulated during egg gametogenesis (75). This increase may suggest that energy-rich unsaturated, essential fatty acid reserves are being preferentially converted to regulate physiological functions such as wound healing, detoxification and vasodilation (76).

### Gangliosides, amino acids, and peptides

Gangliosides, sialic acid-containing glycosphingolipids, play a vital role in neural development and supporting immune responses against disease (77). Gangliosides, including GM3-type, have been isolated from seastars and have been implicated in neural regeneration (78–80). It is not clear why there was drastic reduction in the levels of GM3-type ganglioside in food-deprived COTS (Table S1), although the likely explanation is that it is further catabolised by sequential removal of sugar units in the oligosaccharide group to ceramides to unlock energy reserves. Amino acids may be utilized for protein synthesis during starvation as well as to provide an additional energy source. In most cases the abundance of amino acids decreased in starved COTS, however, arginine was significantly increased. In the *A. rubens*, arginine plays a role in the biosynthesis of nitric oxide which in turn mediates the relaxation of the cardiac stomach (81). In the absence of food, the biosynthesis of nitric oxide is possibly slowed, resulting in the accumulation of arginine.

## Conclusions

In this study, we provide an expansive list of metabolites identified in the COTS RNC, from which we have focused on a number of well-known metabolites involved in animal reproduction and food regulation (82–85), including small molecule neurotransmitters. It has been assumed that prolonged food deprivation is a possible causation for a COTS population collapse (86), possibly a result of increased vulnerability to disease and down-regulated reproductive processes. Our metabolomic data shows a clear distinction between each experimental group's metabolic makeup, including the relative abundance of small molecule neurotransmitters that are known to be key regulators of neural and endocrine events in other animals. Information derived from this study provides a platform for in-depth studies into neural chemical signaling in COTS, and other echinoderms, which may provide a capacity for targeted animal manipulation strategies. Identification of neurotransmitter biosynthetic enzymes and spatial analysis of their products (in this study GABA, serotonin and histamine) is an important step toward defining the molecular genetic markers that may contribute to research into the neuroanatomy of regeneration, autotomy, and the propensity for population explosions and subsequent collapse.

## Author contributions

MS and UB carried out the sample collection, laboratory work for the molecular biology and chemistry components, data analysis, bioinformatics, schematic and figure preparations. MM carried out L-glutamic acid bioassays. MH assisted with animal husbandry. AE, CM, and SC assisted with data analysis and all authors contributed to manuscript preparation.

### Conflict of interest statement

The authors declare that the research was conducted in the absence of any commercial or financial relationships that could be construed as a potential conflict of interest.

## Supplementary material

Figure S1 LC-MS total ion chromatograms [(Retention time (R*t*) 2-24 min)] of Crown-of-Thorns Seastar (COTS) radial nerve cord.

Figure S2 Principal component analysis (PCA) scores plot, PC1 (t (1)) vs. PC2 (t (2)) showing the variation in the profiles of metabolites from four experimental groups: male control (MF, green), male food-deprived (MFD, blue), female control (FC, black) and female food-deprived (FFD, red). Each symbol represents one biological replicate from each individual experimental group described by all detected metabolites.

Figure S3 Immunofluorescent localization controls using serotonin neurotransmitters in the Crown-of-Thorns Seastar radial nerve cord (x10 magnification). **(A-C)** Immunofluorescence analysis with DAPI nuclear stain and using anti-serotonin (5-HT) with images overlaid, respectively. **(D–F)** Negative immunofluorescence antibody analysis with DAPI nuclear stain and using anti-serotonin preabsorbed with serotonin (568 nm), with images overlaid, respectively. **(G–I)** Negative immunofluorescence antibody analysis with DAPI nuclear stain and using anti-serotonin preabsorbed with serotonin (488 nm) and images overlaid, respectively. Scale bars = 50 μm.

File S1 Enzymes identified within Crown-of-Thorns Seastar genome and transcriptome database that are putatively involved in biosynthesis of GABA, histamine, serotonin, melatonin, epinephrine, and octopamine.

Table S1 Details of metabolites identified from Crown-of-Thorns Seastar radial nerve cord.
