# Supplementary figures and images for: Differences in Small Molecule Neurotransmitter Profiles From the Crown-of-Thorns Seastar Radial Nerve Revealed Between Sexes and Following Food-Deprivation
Source: Front Endocrinol (Lausanne). 2018 Oct 15;9:551. doi: 10.3389/fendo.2018.00551 (PMC6196772; doi:10.3389/fendo.2018.00551)

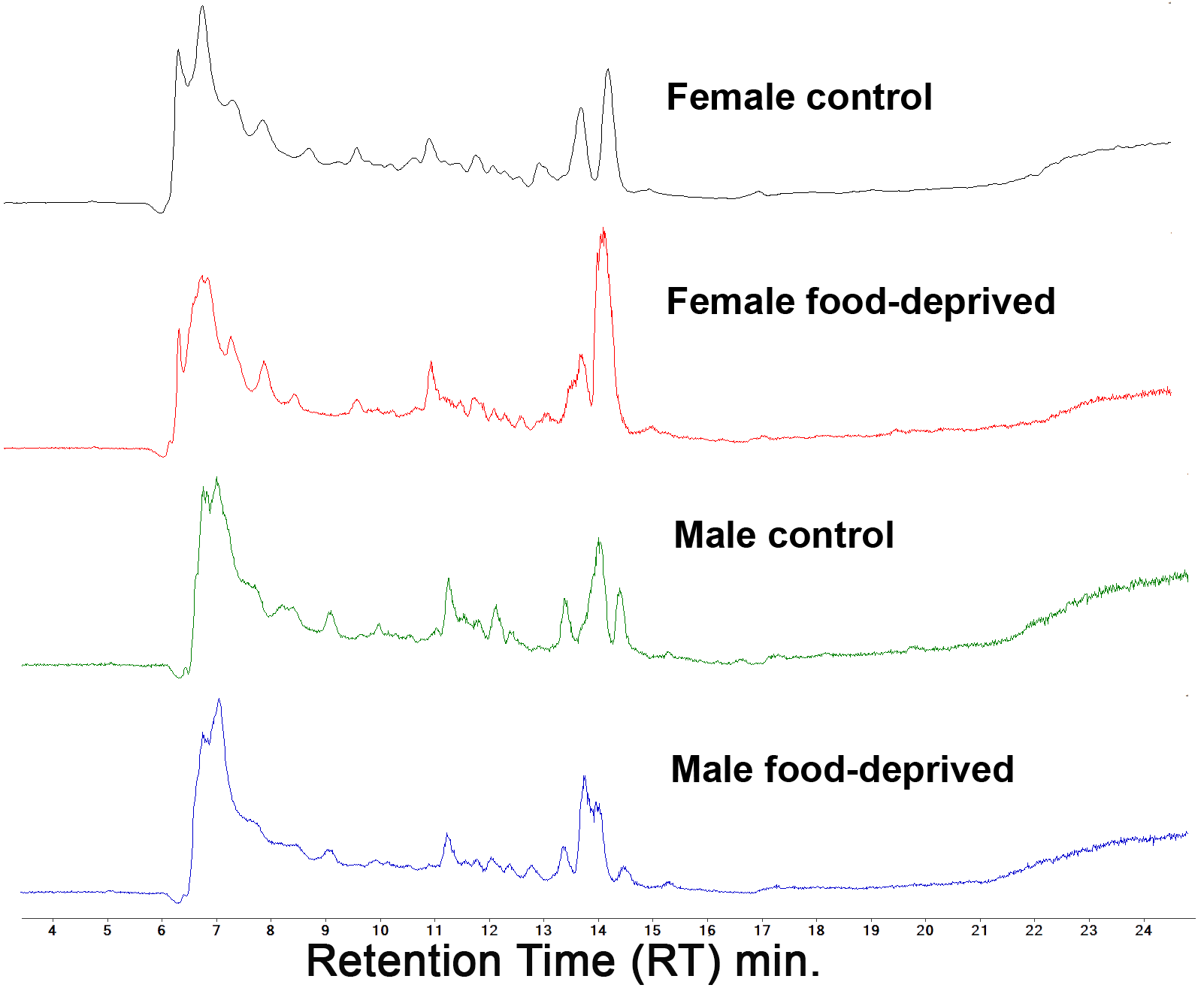

Supplement: Figure S1 — LC-MS total ion chromatograms [(Retention time (Rt) 2-24 min)] of Crown-of-Thorns Seastar (COTS) radial nerve cord. [file Image_1.TIF]

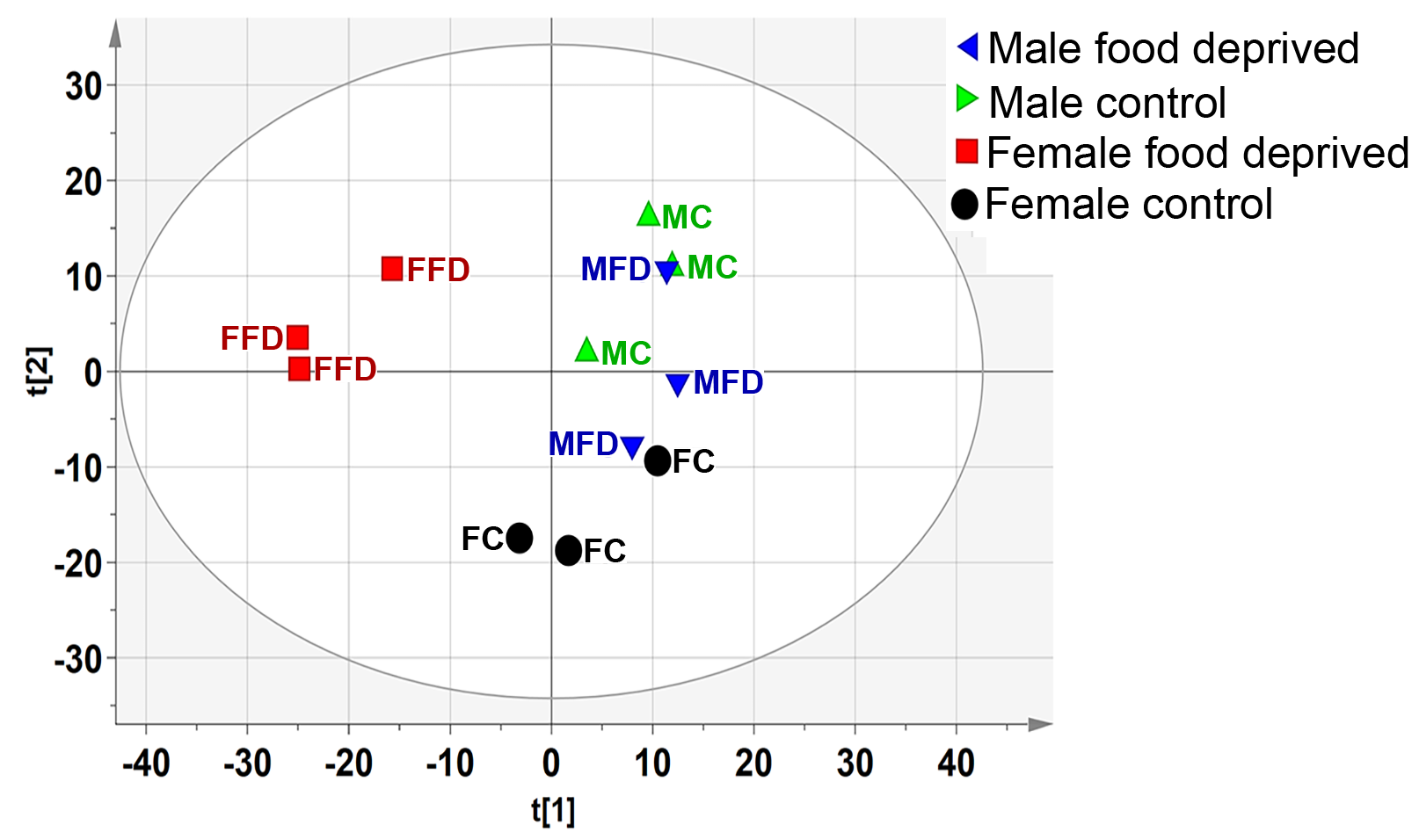

Supplement: Figure S2 — Principal component analysis (PCA) scores plot, PC1 (t (1)) vs. PC2 (t (2)) showing the variation in the profiles of metabolites from four experimental groups: male control (MF, green), male food-deprived (MFD, blue), female control (FC, black) and female food-deprived (FFD, red). Each symbol represents one biological replicate from each individual experimental group described by all detected metabolites. [file Image_2.TIF]

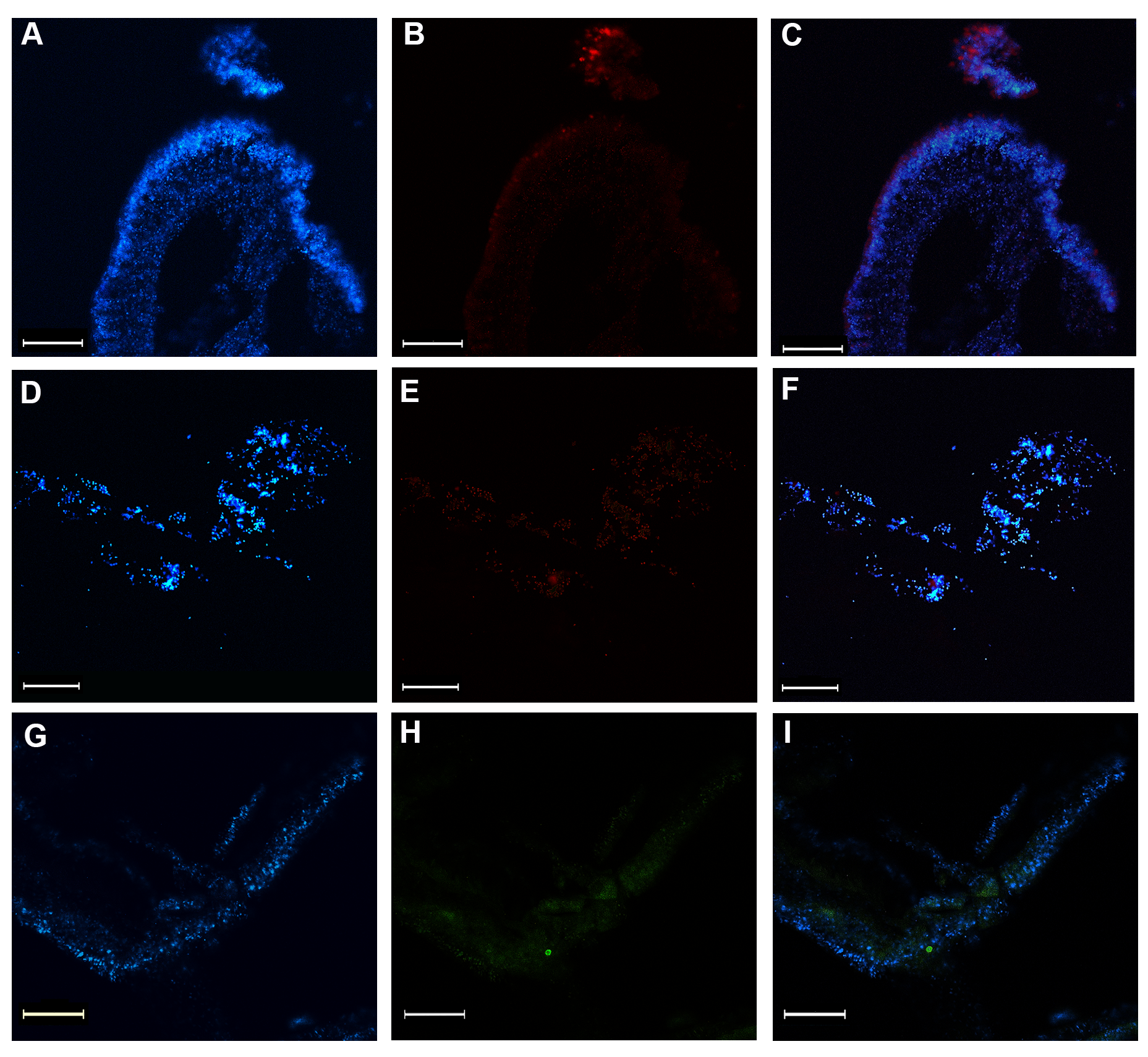

Supplement: Figure S3 — Immunofluorescent localization controls using serotonin neurotransmitters in the Crown-of-Thorns Seastar radial nerve cord (x10 magnification). (A-C) Immunofluorescence analysis with DAPI nuclear stain and using anti-serotonin (5-HT) with images overlaid, respectively. (D–F) Negative immunofluorescence antibody analysis with DAPI nuclear stain and using anti-serotonin preabsorbed with serotonin (568 nm), with images overlaid, respectively. (G–I) Negative immunofluorescence antibody analysis with DAPI nuclear stain and using anti-serotonin preabsorbed with serotonin (488 nm) and images overlaid, respectively. Scale bars = 50 μm. [file Image_3.TIF]
