# Supplementary material for: Differences in Small Molecule Neurotransmitter Profiles From the Crown-of-Thorns Seastar Radial Nerve Revealed Between Sexes and Following Food-Deprivation
Source: Front Endocrinol (Lausanne). 2018 Oct 15;9:551. doi: 10.3389/fendo.2018.00551 (PMC6196772; doi:10.3389/fendo.2018.00551)
Supplement: File S1 — Enzymes identified within Crown-of-Thorns Seastar genome and transcriptome database that are putatively involved in biosynthesis of GABA, histamine, serotonin, melatonin, epinephrine, and octopamine. [file Data_Sheet_1.docx]

Enzymes identified within COTS genome and transcriptome database required for serotonin, GABA and histamine biosynthesis

Serotonin

>Tryptophan_hydroxylase (Oki.6.202)

MDGEPATKLRRLETNGSVDDAGKGNSEVRGENGRTSLMFGLKEEVGILARTLKLFEEHGINLSQIESRPSKGDEGSYEFLVICEEKADKLDTVLDKLKEEATYVHVLSRSQEENTIPWFPRRIKDTDEFANNILSYGAELDADHPGFTDPVYRARRKEFADIAFNYKHGTPIPHVTYTADEIKTWGTMFRELTKLFPTHACREFNHIFPLLVENCGYREDNIPQLEDVSNFLKDCTGFTLRPVAGLLSPRDFLAGLAFRVFHSTQYIRHGSNPWYTPEPDVCHELIGHVPLFADPSFAQFSQEIGLASLGAPDEYIEKLSTCYWFTVEFGMCIQNGQRKAYGAGLLSSYGELQYCLTDKPEFKPFNPAKTAVQKYPISSFQPMYFVAESFEDAKDKMRQFASIIPRPFSVRYNAYTQSVEVLEKKTQLKELAQSIREPMQTVTNLNMTALSPPPGTLCRWYTLLAGRGARRQSRGPEHLDRCCQQSLLNTPKFSTDTSWDIAPRNFKRDLNILIDAVNKVS

>aromatic_L-amino_acid_decarboxylase (Oki.167.44)

MDGETFCRRAAEMADFITKYLRDIKEYRVMPAVSPGYLHRLLPREAPWKPESWESIMSDVEDTILPGMTHWQHPGFHAYFPAGNSYPSILADMLSDGLGCIGFSWAASPAMTELETLMVDWLGRMMGLPKEMLPYTEGGTGSGVMQGSASECTLVCMLAARAKALKELKEVYPDEEDCVLLSKLVAYFSEEAHSSVEKAANIAFVKKRKLLTDDHYAFRGETLRKAIQIDVEKGLVPFFVCATIGTTGVCACDDVEGLGRVCSERNLWLHVDGAYGGNALICPEFRYLLRGFQYVTSFNFNPNKWMMVNFDCSVMWVRDKHALTNALTVNPVYLQHDNEAHSIDYRNWTIPLSRRFRSLKLWFVIRTYGVEGLQRYIRSHVVMAKRFETLVQQDDRFEVLGDVIFGLVCFRLKGQNNLTETLLRRINQSGKAHMVPTSLRGKFVIRFAVCHPDPTEEHIDQTWAVVTDHAERVLFTSEVAVEKFRRLLTKYKENKGRRSTRILGELKKNVREPVQVQAKRKFKL

GABA

>Glutamate_decarboxylase (Oki 2.103)

MISDTNLIGTVSEESEFLQVENNPEDAENQDVSEDRTTTGNCHPNQAKNAANPPAPRRRVIGDRPRLVDRTVSEGQLLVPKKNQRQHEDDDQPDFFEFRAKDLLPCKGSTGLTTKFLKEVTEVILEYVQATFDRSSKILEFHHPNELRERLHLEIPDKAENLDQILQDCQNTLKYCVHTGHPRFFNQLSTGLDIISLAGEWLSAAANTNMFTYEIAPVFTLMERAILKKMREIIGYKDGDGIFAPGGAVSNLYAVLCARHKYVPSCKRRGLSGAPNLVLFTSEHSHFSLKRAAAILGIGTDNVVYIDTDNRGKMNLEDLENKIQQATARGDKPFFVNATAGTTVLGAFDPLNAVADLCEKYNLWMHVDAAWGGGVLMSRKHRHKMDGIHRADSVTWNPHKLMGVLLQCSAVLLKEDCILEDCNSMRAPYLFQQDKHYDVSYDTGDKTIQCGRHVDVFKFWLMWRAKGTRGFENHVNKLFDLAQYLLEKLKSREGFKLVYDKPEHTNVCYWYIPPSLRSMEEGREKQLLLHKVAPAIKARMMAAGTLMVGYQPLGNRVNFFRMVFSNPAATKADVDFLLDETERLGRDL

>Trypophan_hydroxylase (Oki.6.202)

MDGEPATKLRRLETNGSVDDAGKGNSEVRGENGRTSLMFGLKEEVGILARTLKLFEEHGINLSQIESRPSKGDEGSYEFLVICEEKADKLDTVLDKLKEEATYVHVLSRSQEENTIPWFPRRIKDTDEFANNILSYGAELDADHPGFTDPVYRARRKEFADIAFNYKHGTPIPHVTYTADEIKTWGTMFRELTKLFPTHACREFNHIFPLLVENCGYREDNIPQLEDVSNFLKDCTGFTLRPVAGLLSPRDFLAGLAFRVFHSTQYIRHGSNPWYTPEPDVCHELIGHVPLFADPSFAQFSQEIGLASLGAPDEYIEKLSTCYWFTVEFGMCIQNGQRKAYGAGLLSSYGELQYCLTDKPEFKPFNPAKTAVQKYPISSFQPMYFVAESFEDAKDKMRQFASIIPRPFSVRYNAYTQSVEVLEKKTQLKELAQSIREPMQTVTNLNMTALSPPPGTLCRWYTLLAGRGARRQSRGPEHLDRCCQQSLLNTPKFSTDTSWDIAPRNFKRDLNILIDAVNKVS

Histamine

>Histidine_decarboxylase (Oki.205.22)

MDALEYQRRGKEVVDFIVEYLTTIRTRRTFPDVQPGYMKRLVPDVAPQEGEKWEDIFKDVERVILPGVTHWQSPHMHAYFPALNSFPSLLGDMVADAISCLGFTWASSPACTELEIIVMDWLGAMIGLPPCFMHRNENGKGGGVLQGTLSEATLVAIFAARYRAINRERERENLSDDISDGAICSRLVVYCSDQAHSSVEKNALISLVKLRRIQSDERYSLRGSTLRKAIDEDEKKGLIPFFVCATLGTTGVCAFDNLDEIGEVCKEKKLWLHVDAAYAGTAFLCPEYRTHLHGVDKADTFAFNPSKWMMVHFDCTALWVKDREVLENTFCVNPLYLKHEKQGMAVDFMNWQIPLSRRFRAIKLWFVIRSFGIKGLQEHVRKGVRLAKYFESLLRTEPIFEIPVECNLSLVVFRLKGKNFLTEELLRRLNATGKLYVVPAAINGLYVIRFSVTSFYTTEEDIQKDWRLICSMGNAVMRCLSPLRRRIASWPAAAAFEARPDVYMALYTESKMAALAIPDEADGYSSDTDGIDLRKSPSLYPRKPFSVQEKLSPESEDNSGEDCPNGACMMNGRSNGLGDEDNKSQDDVFYHPTKGSVSLGGSMPQLNGLSVEVTKSVVVQNNEKKMGLYDIQNGLDDKPTQVSIASRGRFGALSVAKESTYTGVVNICHCSAKEDTNTLEEN
